# Non-invasive brain stimulation for borderline personality disorder: a systematic review and network meta-analysis

**DOI:** 10.1186/s12991-025-00561-1

**Published:** 2025-04-16

**Authors:** Mohamed Ezzat M. Mansour, Khalid Radwan Alsaadany, Mohamed Awad E. Ahmed, Ahmed Ezzat Elmetwalli, Ibrahim Serag

**Affiliations:** 1 Faculty of Medicine, Zagazig University, Zagazig, Egypt; 2 Faculty of Medicine, Mansoura University, Mansoura, Egypt; 3 Faculty of Medicine, Suez University, Suez, Egypt; 4 Faculty of Medicine, Misr University for Science and Technology, Giza, Egypt

**Keywords:** Borderline personality disorder, BPD, Non-invasive brain stimulation, Transcranial magnetic stimulation, Transcranial direct current stimulation

## Abstract

**Introduction:**

Borderline Personality Disorder (BPD) is a complex neuropsychiatric condition characterized by four main symptom domains: emotion dysregulation, behavioral dysregulation, self-image disturbances, and interpersonal instability. While psychotherapy remains the primary treatment, there is a need for additional effective interventions. Given the neuromodulatory effects of non-invasive brain stimulation (NIBS) techniques, such as transcranial magnetic stimulation (TMS) and transcranial direct current stimulation (tDCS), these methods may hold potential for addressing BPD symptoms.

**Methods:**

A systematic review and network meta-analysis were conducted following PRISMA guidelines. A literature search (PubMed, Scopus, Web of Science, Cochrane CENTRAL) identified comparative studies assessing the effects of NIBS in BPD. The primary outcome was impulsivity, measured by the Barratt Impulsivity Scale (BIS-11). Secondary outcomes included Depressive symptoms, which were evaluated using different scales such as the Hamilton Depression Rating Scale (HAMD) and the Beck depression Inventory (BDI) scale, and anxiety symptoms were evaluated using the Hamilton Anxiety Rating Scale (HAMA).

**Results:**

Five studies with a total of 103 patients were included. Regarding impulsivity, tDCS 2 mA showed a significant reduction compared to the control group (MD = -11.67, 95% CI [-21.44, -1.90]). For depressive symptoms, TMS 20 Hz ranked highest (SMD = -1.97, 95% CI [-3.51, -0.43]), followed by tDCS 2 mA (SMD = -1.65, 95% CI [-2.97, -0.34]). In terms of anxiety, both TMS 5 Hz (MD = -12.29, 95% CI [-24.57, -0.01]) and tDCS 2 mA (MD = -11.81, 95% CI [-17.39, -6.23]) showed significant differences.

**Conclusion:**

Preliminary evidence suggests potential efficacy of non-invasive brain stimulation for BPD, with well-tolerated side effects with well-tolerated side effects. Although there are noticeable statistically significant differences between the interventions and control groups, the results are inconclusive due to the small sample.

**Supplementary Information:**

The online version contains supplementary material available at 10.1186/s12991-025-00561-1.

## Introduction

Borderline Personality Disorder (BPD) is a complex and challenging mental health condition characterized by a pervasive pattern of instability in affect, emotion, and marked impulsivity [1, 2]. One of the main symptoms of BPD is emotion dysregulation, which significantly contributes to its psychopathology. Individuals with BPD often experience unstable moods, impulsive behaviors, and turbulent interpersonal relationships [3]. The etiology of BPD is multifactorial and heterogeneous, with current theories favoring a stress-diathesis model. This model posits that BPD arises from an interaction between genetic predispositions and adverse childhood experiences, such as sexual abuse or neglect [4, 5]. The disorder imposes a substantial burden on patients, their families, and healthcare systems. Despite being historically viewed as untreatable, advancements in our understanding and management of BPD have facilitated earlier diagnoses and improved treatment outcomes [6]. In the United States, BPD affects approximately from 1.4 to 5.9% in community samples [7]. The disorder is notably associated with high rates of suicidal behavior and self-harm, with an estimated 10% of individuals with BPD ultimately succumbing to suicide [8]. BPD imposes a significant economic burden on society due to the extensive utilization of treatment services. However, studies indicate that treating BPD with evidence-based psychotherapy results in a mean cost saving of USD $2,987.82 per patient per year [9]. 

To understand the mechanism of BPD, many neuroimaging studies have been done to explore the mechanism leading to the manifestation of BPD. Functional neuroimaging and neuropsychology studies have identified a dysfunctional frontolimbic network that may be involved in clinical symptoms, including emotional instability and impulsivity [10–14]. These studies consistently illustrate dysfunctions in prefrontal regions, including the orbitofrontal cortex, ventromedial prefrontal cortex, and dorsolateral prefrontal cortex (DLPFC). Additionally, BPD patients exhibit hyperactivity of the amygdala when processing emotional stimuli, coupled with reduced functional activity in prefrontal structures responsible for emotional regulation [15–18]. A recent meta-analysis concluded that BPD patients’ impairments in the cognitive control of negative stimuli are presumably the result of blunted activity of the DlPFC along with enhanced activation of the limbic system [19]. Another meta-analysis of functional neuroimaging studies exploring neural correlates of negative emotionality in BPD showed that patients with BPD display enhanced activity in the insula and posterior cingulate cortex, but reduced activity in a network of regions including the medial PFC, subgenual anterior cingulate cortex, and DLPFC [20–22]. 

BPD remains a challenge to treat and manage. From a therapeutic perspective, although no specific pharmacological treatment has been licensed, about 75% of BPD patients regularly take psychotropic medication [23, 24]. Pharmacological treatment results remain equivocal, and these interventions show some promise in addressing impulsive behaviors, but their efficacy remains uncertain [25, 26]. Psychological interventions, particularly psychotherapy, are the first-line treatment for BPD, as recommended by the **NICE guidelines**. Pharmacological treatment is generally reserved for cases of acute decompensation or comorbid psychiatric conditions, although a significant proportion of BPD patients receive multiple psychotropic medications, (rTMS) and transcranial Direct Current Stimulation, (tDCS) - represent ground-breaking tools with a wide range of diagnostic, neurophysiological, and therapeutic applications, reversing maladaptive neurocircuits, inducing changes in neural tissue and improving abnormal neural connectivity in BPD. tDCS represents one of the new ways to treat patients with BPD. A simple and presumably effective way to increase cortical brain activity [27]. tDCS is a promising, low-risk, non-invasive neuromodulation technique that relies on the application of a weak direct current of 1–2 mA to generate regional changes in cortical excitability, which, depending on the duration and the polarity, can last for several minutes up to a few hours after stimulation [28, 29]. Experimental studies show that excitatory DlPFC stimulation improves cognitive control of aversive stimuli in both healthy individuals and BPD patients [30]. rTMS is a potentially innovative method in the treatment of BPD. Some studies targeted the prefrontal cortex because it is hypothesized that high-frequency rTMS (typically ≥ 5 Hz) can increase prefrontal excitability and, subsequently, prefrontal-limbic connectivity, whereas low-frequency rTMS (≤ 1 Hz) may exert inhibitory effects on cortical hyperactivity [31–33]. The DMPFC-rTMS has been proposed to enhance cognitive control and reduce impulsivity in patients with BPD.

Given the limitations observed in the current literature, future studies should aim to establish more standardized protocols for Non-Invasive Brain Stimulation in BPD. Specifically, randomized controlled trials (RCTs) with larger sample sizes, standardized stimulation parameters, and long-term follow-up assessments are needed to determine the sustained effects of interventions such as repetitive rTMS and tDCS. Moreover, neuroimaging-guided stimulation protocols could enhance precision in targeting dysfunctional neural circuits, such as the frontolimbic network, which is implicated in impulsivity and affective dysregulation in BPD. Combining NIBS with established psychotherapeutic approaches may also improve clinical outcomes, leveraging the potential synergistic effects of both interventions [30]. Addressing these research gaps will be critical to refining the therapeutic role of NIBS in BPD and guiding its clinical application.

Our systematic review and network meta-analysis aims to synthesize evidence on the efficacy and safety of Non-Invasive Brain Stimulation (e.g., rTMS, tDCS) in reducing core BPD symptoms, including emotional dysregulation and impulsivity, Compare the effects of different NIBS modalities (e.g., high- vs. low-frequency rTMS, TDCS) and highlight gaps in the literature to guide future research.

In this context, our study aims to provide a comprehensive synthesis of the available evidence on the safety and efficacy of NIBS for BPD, using a systematic review and network meta-analysis approach. By comparing different NIBS modalities, we seek to identify the most effective stimulation parameters and assess their impact on key clinical outcomes, including emotional regulation, impulsivity. Our findings will contribute to the development of evidence-based recommendations for the use of NIBS in BPD treatment and inform future research directions in this field.

## Methods

The PRISMA guidelines were used in this systematic review and network meta-analysis [34]. We established this study according to the fundamentals of the Cochrane Handbook of Systematic Reviews of Interventions [35]. This study was previously registered on the International prospective register of systematic reviews PROSPERO (CRD42024538574).

### Criteria for considering studies in this review

Studies satisfying the following inclusion criteria were included in the systematic review:

*Population*: studies on adult patients with a primary diagnosis of BPD according to DSM-IV / DSM-V criteria.

*Intervention*: rTMS or tDCS, followed for at least four weeks of stable pharmacological treatment. All frequencies (Hz) were eligible.

*Comparator*: studies where the control group received a Sham-control.

*Outcome*: Studies reporting at least one of the following measures—Depression or Impulsivity, with the latter assessed using the Barratt Impulsivity Scale (BIS).

*Study design*: randomized controlled trials (RCTs).

We excluded articles that were case reports/case series, thesis, conference abstracts, animal studies, secondary studies, and studies investigated other psychological disorders (Psychosis, drug abusers, and Bipolar disorders) other than BPD where the patients were not diagnosed according to the previously mentioned BPD criteria. Patients with serious or uncontrolled comorbidities, such as pregnancy, and contraindication to (TMS/tDCS) were excluded from completing the experiment in each study design.

### Literature search keywords

We conducted a comprehensive search using various databases such as PubMed, Scopus, Cochrane CENTRAL, Web of science, and EBESCO for relevant studies until the 13th of June. The search strategy was:

((Borderline Personality Disorder) OR (Personality Disorder)) AND ((Non-Invasive brain stimulation) OR (Transcranial Magnetic Stimulation) OR (TMS) OR (TansCranial Direct Current Stimulation) OR (tDCS) OR (Theta Burst Stimulation) OR (TBS) OR ( Transcutaneous Vagal Nerve Stimulation) OR (tVNS) OR (Transcranial Alternating Current Stimulation) OR (tACS)).

### Screening and study selection process

The process of literature search and screening were done separately by two authors (MEM and KRE). Eligibility screening was done using Rayyan [36]. Studies screening were ongoing in two levels. The first level was screening the title/abstract to ensure matching for the inclusion criteria. In the second level, we checked the full text for eligibility to our meta-analysis criteria.

### Data extraction

All authors participated in the data extraction independently using an online data extraction form. The extracted data consisted of 4 domains: [1] study characteristics [2], characteristics of the study population [3], risk of bias domains, and [4] study outcomes. Data was exported as a Microsoft Excel sheet.

### Assessment of risk of bias in included studies

We assessed the quality of each included study using the Cochrane risk of bias (ROB) tool [37]. The Cochrane ROB tool was designed to assess the probability of bias based on 7 domains: (a) random sequence generation, (b) allocation concealment, (c) blinding of the investigators and patients, (d) blinding of the outcome assessors, (e) incomplete outcome data, (f) selective outcome reporting, and (g) other sources of bias. After careful screening of the structure and data presented in the published RCTs. In each domain, each study was stamped as " low risk of bias “, " high risk of bias “, or " unclear”.

### Measures of treatment effect

The primary outcome measurement, in studies assessing efficacy of NIBS on BPD, was the Barratt Impulsiveness Scale. It is a widely recognized and influential tool in the development of current theories on impulse control. It consists of 30 items that assess three key areas: attentional impulsivity, motor impulsivity, and non-planning impulsivity. The scores range from 30 (low impulsivity) to 120 (high impulsivity) [38]. 

**The secondary outcome** measurements were Depression, HAM-A, CGI-BPD, and BPD Severity.

HAM-A (Hamilton Anxiety) scale, which is a 14-item questionnaire used to rate the severity of a patient’s anxiety. The score takes the range of 0 (the best) to 56 (the worst) [39]. 

Depression was measured by different scales:

1. BDI (Beck depression Inventory) scale, which is a 21-item scale designed to detect the presence of depression and to measure severity of depression. The least takes the range from (0 to 13) and the most severe (29 to 63) [40].
2. MADRS (Montgomery–Asberg Depression Rating Scale) which is a 10-items questionnaire used to measure the severity of depression and the efficacy of antidepressant treatment, overall score thus ranges from (0 to 60) [41].
3. HAM-D (Hamilton Depression Rating scale). HAM-D contains a 17-item questionnaire used to measure the severity of depression. Normal (0–7), and scores over 24 indicate that the symptoms of depression are severe [42].

The clinical global impression scale for borderline personality disorder patients (CGI-BPD) is a modified version of the Clinical Global Impression (CGI) scale, specifically created to evaluate the severity and changes after treatment in patients with BPD. It includes 10 items that assess the nine key psychopathological aspects of BPD, along with an overall global score. The score takes the range of 1 (the best) to 7 (the worst) [43]. 

BPD severity outcomes: 1- Zanarini Rating Scale for Borderline Personality Disorder (ZAN-BPD) evaluates various aspects of BPD symptoms by assessing changes in symptoms over time. It consists of 10 items and each item is rated on a scale from 0 (no symptoms) to 4 (severe symptoms) [44]. 2- Borderline Personality Disorder Severity Index (BPDSI) is used for assessing the severity of BPD. It consists of 70 items and each item is rated on a scale from 0 (no symptoms) to 4 (severe symptoms) [45]. 3- Borderline Evaluation of Severity over Time (BEST) is designed to monitor changes in symptoms and treatment effects across different time points. It consists of 27 items and each item is rated on a scale from 0 (no symptoms) to 4 (severe symptoms) [46]. 

### Data synthesis

For the network meta-analysis, we used Metainsight software version 15.4. We conducted a network meta-analysis according to frequentist framework. The MD (Mean difference) was adopted as the effect estimate with a 95% confidence interval (CI). All safety outcomes, dichotomous data from prospectively designed studies, were reported as risk ratio (RR) between the interventions and control group. The fixed effect model was applied for outcomes with consistent results, while the random effect model was applied for outcomes with significant heterogeneity. We considered the nearest time point through the included studies for the primary analysis in the case of multiple time points.

### Assessment of heterogeneity

The Chi-square test (Cochrane Q test) was used to evaluate Statistical heterogeneity among studies. Then, the chi-square statistic, Cochrane Q, was used to calculate the I-squared according to the equation: I^2^$\:=\left(\frac{\mathbf{Q}-\mathbf{d}\mathbf{f}\:\mathbf{Q}}{\mathbf{Q}}\right)$ 𝑥100%. The significant heterogeneity was considered when the Chi-square P value is less than 0.1. sensitivity analysis and sub-group analysis were performed to resolve heterogeneity. Heterogeneity in the forest plots was determined through visual inspection, while the I^2^ and chi-square (χ2) tests were employed to quantify it. The χ2 test was used to examine the presence of significant heterogeneity, and if heterogeneity was detected, it was measured using the I^2^ test. The interpretation of the I^2^ test follows the standards outlined in the Cochrane Handbook for meta-analysis. According to these guidelines, an I^2^ value of 0–40% may not be considered significant, 30–60% may indicate moderate heterogeneity, 50–90% may suggest substantial heterogeneity, and 75–100% may indicate significant heterogeneity.

### Certainty of evidence

The Grading of Recommendations, Assessment, Development, and Evaluations (GRADE) approach has been used to rate the power of the evidence for each outcome based on the risk of bias, imprecision, indirectness, inconsistency, and publication bias. We assessed the certainty of evidence using the GRADE approach [47]. 

## Results

### Results of literature search

**Fig. 1:**
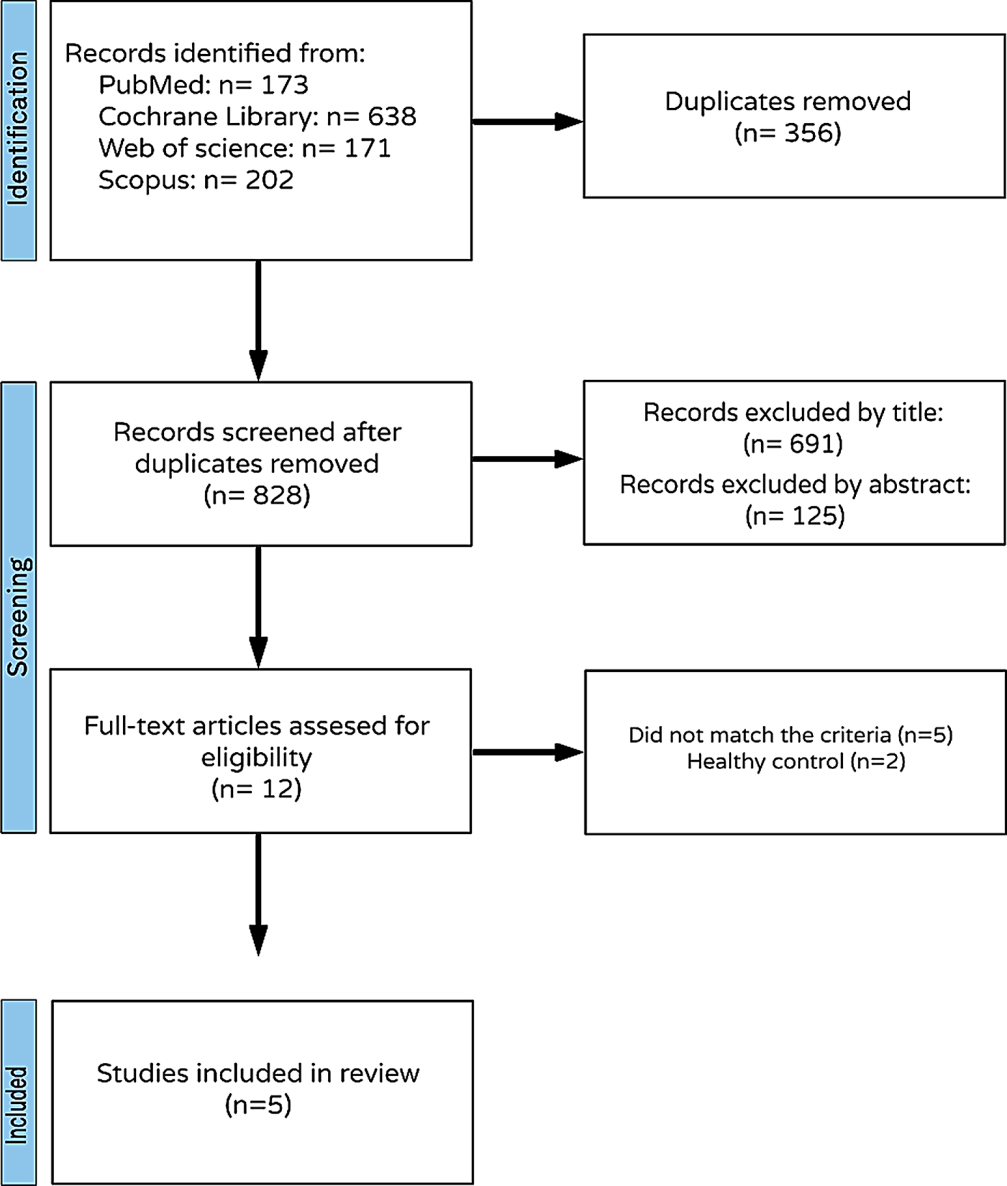
The PRISMA flow diagram

A total of 1184 unique articles were included in the process of literature search by two independent authors (MEM and AEE). Of them, 356 were identified as duplicates by Rayyan. Twelve unique full texts were reviewed and screened for the eligibility criteria. The included RCTs in this meta-analysis were five. The PRISMA flow diagram of the study selection process is shown in Fig. 1.

### Characteristics of included studies

A total of 103 patients were represented in 5 articles. Patients were assigned to receive (TMS /tDCS) or SHAM for at least a session/day. The recruited patients with age range (18–65) years, had a primary diagnosis of BPD according to DSM-V or DSM-IV. The duration of each trial ranged from 3 weeks to 3 months and the dose from (5–20 Hz). A summary of the included studies is shown in Table 1, and the baseline characteristics of their populations are shown in Table 2.

**Table 1:** Summary of the included studies

Study ID	Location	Sample size	intervention	Control	Population	Duration
Reyes-Lopez 2018 [33]	Mexico	29	TMS 5 Hz on left DLPFC TMS 1 Hz on right DLPFC		Adult patients (18–45 years) who primarily diagnosed by BPD according to DSM-IV and a score > 8 Borderline Diagnostic Interview Revised (DIB-R).	15 sessions in 3 weeks
Calderon-Moctezuma 2021 [48]	Mexico	18	TMS 5 Hz on DMPFC	Sham	Adult patients with Mean age 26.03 + 7.08 years who primarily diagnosed by BPD according to DSM-IV and a score > 7 (DIB-R).	15 sessions in 3 weeks
Feffer 2021 [49]	*Canada*	20	TMS 20 Hz on DLPFC	Sham	Adult patients (18–65 years) who primarily diagnosed by BPD according to DSM-5 and a score > 18 HAM-D	15 daily TMS sessions over 3 weeks, followed by a crossover phase of another 15 daily sessions over 3 weeks.15 sessions in 2 weeks
Cailhol 2014 [50]	France	14	TMS **10 Hz** on DLPFC	Sham	Adult patients (20–45 years) who primarily diagnosed by BPD according to DSM-IV and	10 sessions in 2 weeks
Lisoni 2020 [51]	Italy	30	tDCS 2 mA on DLPFC	Sham	Adults patients (Mean age 40.3 ± 12.8) who primarily diagnosed by BPD according to DSM-5	15 sessions in 3 weeks

**Table 2:** Baseline characteristics of included studies

Study	Intervention	Age, years (Mean, SD)	Sex (% male)	Education (years) (Mean, SD)	BIS-11 (Mean, SD)	HAM-A (Mean, SD)	CGI-BPD (Mean, SD)	Depression (Mean, SD)			BPD Severity (Mean, SD)			
								HAM-D	MADRS	BDI	BEST		ZAN*-BPD*	BPDSI
Reyes-Lopez 2018	TMS 1 Hz	30.9 ± 7.6	8%	N/A	70.2 ± 12.0	20.2 ± 6.8	41.1 ± 4.7	N/A	N/A	30.9 ± 15.6	41 ± 14.5		N/A	
	TMS 5 Hz	29.6 ± 7.8	7%		73.1 ± 14.2	15.5 ± 6.4	40.2 ± 6			31.9 ± 14.6	42.8 ± 9.7			
Calderon-Moctezuma 2021	TMS 5 Hz	24 ± 6.29	28.57%	15.43 ± 5.28	61.43 ± 19	23.71 ± 7.76	40.29 ± 9.75	27 ± 9.34	N/A		44.43 ± 13.07		N/A	
	Sham	28.14 ± 8.31	42.85%	15.29 ± 1.97	64.29 ± 13.07	23.71 ± 4.92	36.71 ± 12.78	25.86 ± 7.05			37.71 ± 8.95			
Feffer 2021	TMS 20 Hz	33.9 ± 9.8	All female	16 ± 2.7	67.1 ± 5.8	N/A		23.1 ± 2.6	N/A				16.3 ± 3.1	N/A
	Sham	29.8 ± 15.4	All female	14.3 ± 3.0	68.0 ± 7.3			23.0 ± 3.7					17.8 ± 3.3	
Cailhol 2014	TMS 10 Hz				N/A				16.4 ± 7.2	N/A				19.9 ± 6.6
	Sham								18.7 ± 14.8					24.06 ± 11.3
Lisoni 2020	tDCS 2 mA	38 ± 10.9	7 (46.7%)	12.6 ± 3.8	76 ± 14.34	25.60 ± 5.96	N/A	16.66 ± 4.27	N/A					
	Sham	42.6 ± 13.6	5 (33.3%)	11.8 ± 11.8	72.33 + 9.67	19.66 + 6.49		14.26 ± 4.89						

*Abbreviations* MD, Mean Difference; SD, Standard Deviation; BIS-11, Barratt Impulsiveness Scale; HAM-A, Hamilton Anxiety Rating Scale; CGI-BPD, Clinical Global Impressions Scale for Borderline Personality Disorder; BIS-11, Barratt Impulsiveness Scale; HAM-D, Hamilton Depression Rating Scale; MADRS, Montgomery-Åsberg Depression Rating Scale; BDI, Beck Depression Inventory; BEST, Borderline Evaluation of Severity Over Time; ZAN-BPD, Zanarini Rating Scale for Borderline Personality Disorder; BPDSI, Borderline Personality Disorder Severity Index

### Risk of bias of included studies

**Fig. 2:**
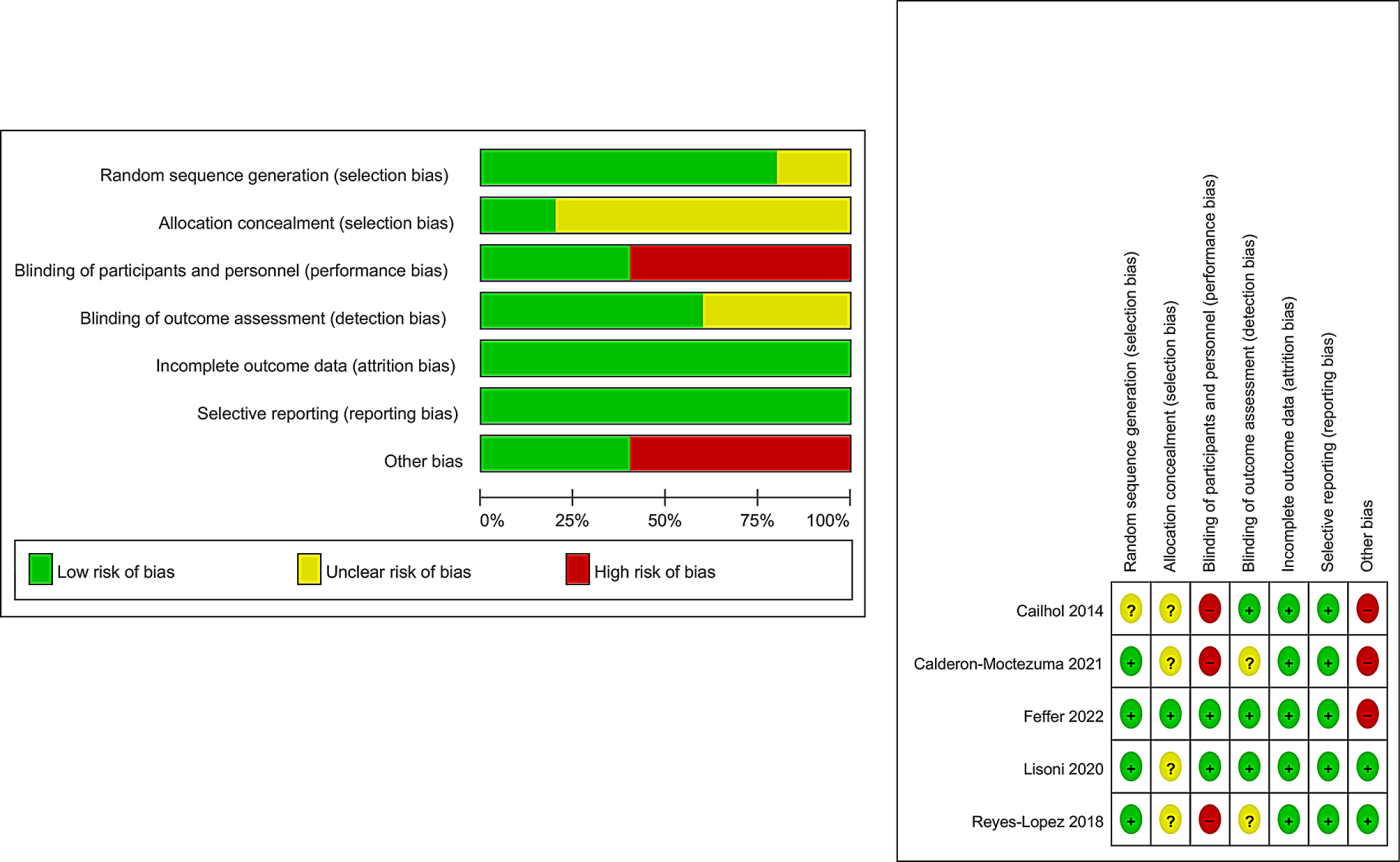
Quality assessment using Cochrane risk of bias tool

The quality of each study was assessed according to the Cochrane Handbook of Systematic Reviews of Interventions by two independent authors (MEM, KRE, MAA). Four of the included studies were rated as High risk of bias and one study was rated as some concerns. A summary of quality assessment domains is shown in **(Figure 2).**

**Fig. 3:**
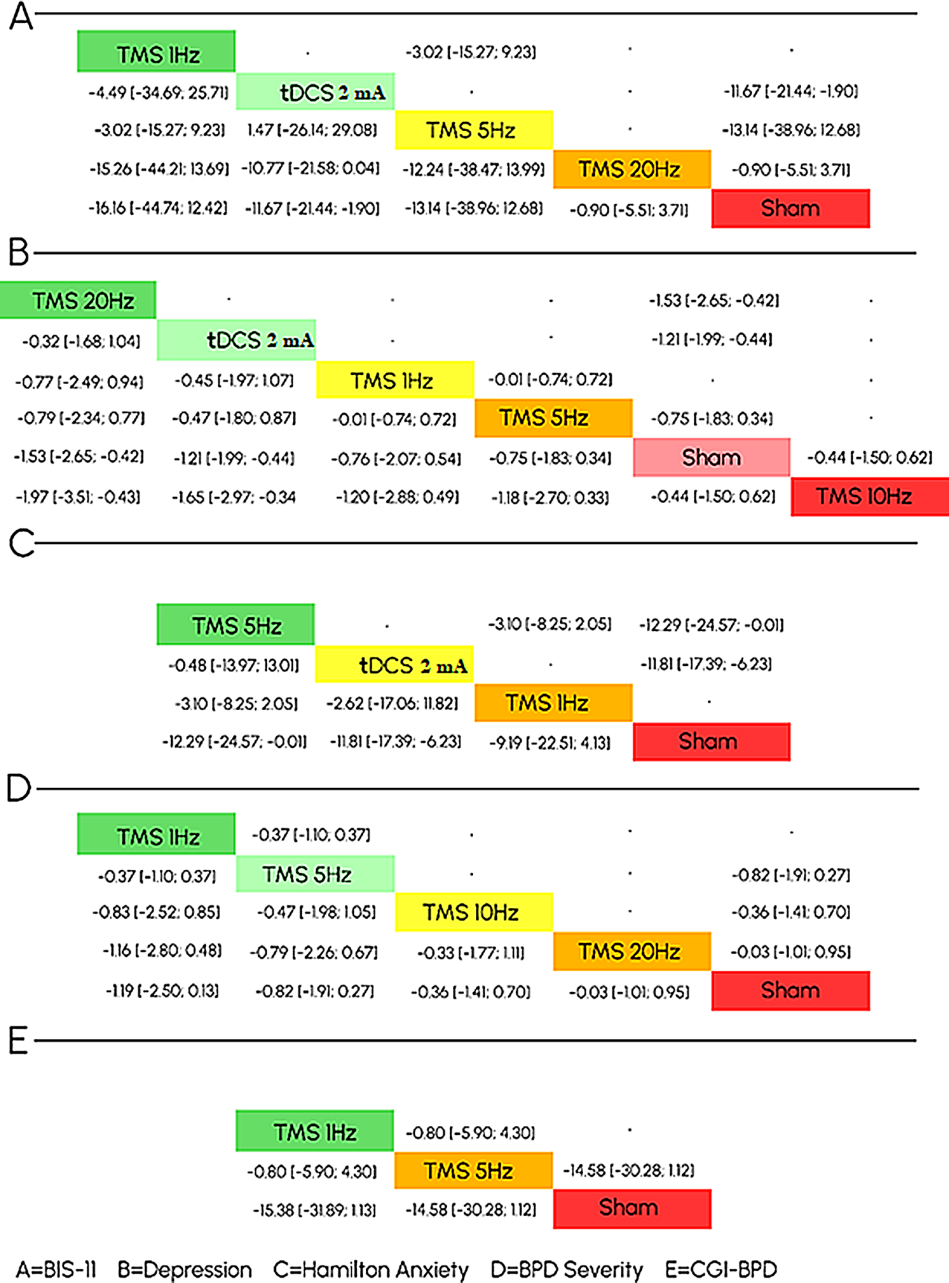
Primary and secondary outcomes

### Primary outcome

#### BIS-11

Four studies comprising 97 patients reported the BIS-11 scale. Figure 3 A showed network estimates of intervention effect on Impulsivity for different techniques compared to sham control. The top two ranked techniques compared to sham control were TMS 1 Hz (MD -16.16, 95% CI [-44.74 to 12.42]) followed by tDCS 2 mA (MD -11.67, 95% CI [-21.44 to -1.90]). Only tDCS 2 mA showed a statistically significant difference. The network plot of Impulsivity is shown in Figure (3 a), each node represents a different technique; the sham control was the most common intervention well-connected with all other interventions directly linked to it. So, it has been used as a reference for comparison.

#### Depression

Five studies comprising 111 patients reported the BIS-11 scale. Figure 3B showed network estimates of intervention effect on Depression for different techniques compared to sham control. The top two ranked techniques compared to sham control were TMS 20 Hz (SMD − 1.97, 95% CI [-3.51 to -0.43]) followed by tDCS 2 mA (SMD − 1.65, 95% CI [-2.97 to -0.34]). Both interventions showed a statistically significant difference. The network plot of Impulsivity is shown in Figure (3b).

### Secondary outcomes

#### Anxiety

Three studies comprising 77 patients reported the BIS-11 scale. Figure 3 C showed network estimates of intervention effect on Depression for different techniques compared to sham control. The top two ranked techniques compared to sham control were TMS 5 Hz (MD -12.29, 95% CI [-24.57 to -0.01]) followed by tDCS 2 mA (MD -11.81, 95% CI [-17.39 to -6.23]). Both interventions showed a statistically significant difference. The network plot of Impulsivity is shown in Figure (3c).

### BPD severity

Four studies comprising 73 patients reported BPD Severity. Figure 3 C showed network estimates of intervention effect on Depression for different techniques compared to sham control. The top two ranked techniques compared to sham control were TMS 1 Hz (SMD − 18.46, 95% CI [-38.30 to 1.38]) followed by TMS 5 Hz (SMD − 12.46, 95% CI [-29.06 to 3.54]). The network model did not show a statistically significant difference among all interventions. The network plot of Impulsivity is shown in Figure (3d).

#### CGI-BPD

Two studies comprising 43 patients reported CGI-BPD. Figure 3 C showed network estimates of intervention effect on Depression for different techniques compared to sham control. The network model did not show a statistically significant difference among all interventions. The network plot of Impulsivity is shown in Figure. Among the nine domains of BPD-CGI, TMS 1 Hz was top-ranked in Abandonment, Anger, Emptiness, Impulsiveness, Paranoid, and Suicidal Ideation. The network model did not show a statistically significant difference among all interventions except Impulsiveness and Paranoid ideation both interventions showed substantial differences, TMS 1 Hz (MD -3.11, 95% CI [-4.37 to -1.85]) followed by TMS 5 Hz (MD -2.71, 95% CI [-3.91 to -1.51]) However TMS 1 Hz was top ranked in for Paranoid ideation, only TMS 5 Hz showed significant difference (MD -2.28, 95% CI [-4.47 to -0.09]). All forest plots of behavioral domains are shown in Supplementary (S1-S9).

#### Safety

It is important to note that no severe side effects were observed in any of the included studies. In Lisonia et *al*, only five patients showed tingling, and itching sensations during stimulation due to tDCS 2 mA. Non-invasive brain stimulation techniques are well-tolerated treatment options for BPD patients.

### The GRADE approach of outcomes

**Table:** 

Outcome	Participants (studies) Follow-up	Risk of bias	Inconsistency	Indirectness	Imprecision	Publication bias	Overall certainty of evidence
BIS-11	Serious ^a^	not serious	not serious	not serious	not serious	none	⨁⨁⨁◯ Moderate
Depression	Serious ^a^	not serious	not serious	not serious	not serious	none	⨁⨁⨁◯ Moderate
HAM-A	Serious ^a^	not serious	not serious	not serious	not serious	none	⨁⨁⨁◯ Moderate
BDA Severity	Serious ^a^	not serious	not serious	not serious	Serious ^b^	none	⨁⨁◯◯ Low
CGI-BPD	Serious ^a^	not serious	not serious	not serious	Serious ^b^	none	⨁⨁◯◯ Low

CI: confidence interval

Explanations:

*a. Presence of studies with a high risk of bias.*

*b. The confidence interval in each intervention is wide which includes clinically important values.*

## Discussion

### Significance of the study

To the best of our knowledge, this is the first systematic review and network meta-analysis comparing the efficacy of TMS and tDCS in BPD. This study evaluates the efficacy of different non-invasive brain stimulation techniques and ranks them by effectiveness across frequencies. Therefore, encourage of conducting future trials involving different techniques such as Theta Burst Stimulation (TBS), Trans Alternating current stimulation (tACS), and Percutaneous Vagal Stimulation.

### Summary of the findings

A total of 103 patients with BPD were included in 5 RCTs comparing TMS, tDCS, and Sham-controlled were evaluated in this study. We found that each outcome demonstrates the highest efficacy of specific frequency each time. So, that means that each frequency intervention is effective in specific domain in the disease. These differences in results could be due to the variation of each technique which can be implicated in the pathophysiology of the disease in different ways.

### BIS

Although TMS 1 Hz did not show a significant difference in the analysis model, it was the top-ranked protocol in this outcome. TMS 1 Hz, there are several explanations which could explain the results. Recently, research indicated that the low-frequency protocol has led to the successful induction of anticorrelated connectivity between the DLPFC and medial prefrontal default mode network (DMN) node [52]. The DMN node is an area found to be active when the individual is engaged in internal thoughts and self-reflection. Also, conducting TMS 1 Hz was found to be associated with a notable alteration in the asymmetry of alpha power towards the right lobes [53]. Alpha power is a neural oscillation (brain wave) measured in different brain activities. It is characterized by waves (8–12 Hz) and it has been suggested that the alpha wave may be involved in the attentional process and prominent during relaxed wakefulness [54].However tDCS 2 mA was ranked secondary in the analysis model, tDCS 2 mA showed significant difference. Conducting tDCS 2 mA plays a crucial role in increasing top-down regulation of the lower-level processes which is the ability of the brain to regulate lower-level cognitive and behavioral processes. In addition, stimulating the right DLPFC by tDCS 2 mA may lead to restoring the interhemispheric disruption [55]. Based on all of that, tDCS 2 mA becomes efficient in reducing impulsivity in BPD. There are some technical factors that could explain the results. In Lopez et *al*, the technicians used the highest angulation angle (150^o^). A higher angulation angle of the device could alter the stimulation focus and depth, potentially affecting the targeted neural circuits involved in impulse control [56]. In addition, authos used high motor threshold (100%) [56]. 

### Depression and anxiety

Depression episodes are the most common comorbidity of BPD. TMS protocols have been widely explored in depression and they are approved for major depressive disorder. The role of TMS 20 Hz in Neuroplasticity Induction leads to long-lasting changes in neural circuits. Also, normalization of the brain activity. All of these can explain the top ranking of TMS 20 Hz for depression symptoms [57, 58]. As TMS 1 Hz in our result is the third-ranked protocol, using the previous suggestion on BPD could be more efficient. As impulsiveness, tDCS 2 mA also achieved the second rank. The efficacy of tDCS 2 mA in depression remains inclusive with unclear evidence in recent years. However, tDCS 2 mA was noticed to stimulate the electric field in the left anterior cingulate cortex, a region of the frontal lobes which implicated in emotion, cognition, and motor control [59]. In addition, tDCS 2 mA might be associated with the regulation of negative effects after DLRPC stimulation [60]. Therefore, future studies should study the actual role of tDCS 2 mA in the pathophysiology of depression, especially in BPD patients. Some technical differences could also give reasons for these results. In Feffer et *al*, authors used the heighst number of pulses per session (3000) over the other trials. Higher pulses could show better antidepressant and antianxious effects rather than lower number of pulses [61].

### CGI-BPD and BPD severity

In our study, no interventions showed significant difference, however TMS 1 Hz was top ranked over TMS 5 Hz in both outcomes. This can be explained by several factors such as the limited number of included studies and patients making it difficult to synthesize significant evidence. So, further studies are needed to show the certain effect of each intervention.

A critical aspect of NIBS interventions in BPD that warrants further exploration is the impact of laterality (right vs. left hemisphere stimulation) on clinical outcomes. While our meta-analysis primarily focused on differences in stimulation protocols, accumulating evidence suggests that right prefrontal cortex stimulation, particularly over the right DLPFC, may be associated with greater improvements in impulsivity control, aligning with neurobiological models that link right hemisphere dysfunction with impaired inhibitory control (Brevet-Aeby et al., 2016). Conversely, left hemisphere stimulation, often targeted for affective symptoms, may exert differential effects on mood regulation. Prior systematic reviews, particularly Lisoni et al. (2022, 2024), have emphasized a symptoms-based approach to NIBS in BPD, highlighting the need for standardized protocols to determine whether the observed effects are attributable to laterality or other stimulation parameters. The heterogeneity in stimulation protocols across the included studies, such as differences in frequency, intensity, session duration, and stimulation site, limits direct comparisons and makes it difficult to disentangle the effects of stimulation site from other variables.

### Strength points and limitations

This network meta-analysis has several strengths. It is the first systematic review and Network meta-analysis studying the different non-invasive brain stimulation techniques for BPD. We used the GRADE qualification to assess the quality of evidence for the efficacy outcomes. The studies spanned a wide geographical range with a variety of populations. The most prominent limitation of the study was the low sample size due to the limited number of the included trials. So, our results are not conclusive. Although there were various including populations, they were all from Western countries with high or middle income. Some studies used different scales for depressive symptoms and BPD severity. We suggest future trials to confirm their effectiveness with all these limitations corrected.

### Implications for future research

This meta-analysis supposes that non-invasive techniques could be beneficial treatment for patients primarily diagnosed with BPD. Since the only available treatment of BPD is psychotherapy with limited treatment options. So, exploring newt interventions becomes essential for a better quality of life for patients and to reduce the economic burden on the health care system. Future trials should consider the other non-invasive techniques such as TBS, tACS, and Vagal that showed promising results in other meant diseases.

## Conclusion

In conclusion, this systematic review and network meta-analysis demonstrate that non-invasive brain stimulation techniques, particularly TMS 20 Hz, TMS 5 Hz and tDCS 2 mA, suggest potential benefits for BPD. These techniques were found to significantly improve symptoms related to impulsivity, depression, and anxiety, with specific frequencies of stimulation proving more effective for outcomes. While the findings highlight the potential of these interventions, the limited sample size and heterogeneity across studies underscore the need for larger, more robust clinical trials to validate these results.

## Electronic supplementary material

Below is the link to the electronic supplementary material.

Supplementary Material 1

## Data Availability

No datasets were generated or analysed during the current study.

## References

[1] Borderline Personality Disorder. Causes, Symptoms & Treatment [Internet]. [cited 2024 Aug 23]. Available from: https://my.clevelandclinic.org/health/diseases/9762-borderline-personality-disorder-bpd

[2] Chapman J, Jamil RT, Fleisher C. Borderline personality disorder. StatPearls. Treasure Island (FL): StatPearls Publishing; 2020. 28613633

[3] Carpenter RW, Trull TJ. Components of emotion dysregulation in borderline personality disorder: a review. Curr Psychiatry Rep. 2013;15(1):335. 23250816 10.1007/s11920-012-0335-2 PMC3973423

[4] Belsky DW, Caspi A, Arseneault L, Bleidorn W, Fonagy P, Goodman M, et al. Etiological features of borderline personality related characteristics in a birth cohort of 12-year-old children. Dev Psychopathol. 2012;24(1):251–65. 22293008 10.1017/S0954579411000812 PMC3547630

[5] Chapman AL. Borderline personality disorder and emotion dysregulation. Dev Psychopathol. 2019;31(3):1143–56. 31169118 10.1017/S0954579419000658

[6] Klein P, Fairweather AK, Lawn S. Structural stigma and its impact on healthcare for borderline personality disorder: a scoping review. Int J Ment Health Syst. 2022;16(1):48. 36175958 10.1186/s13033-022-00558-3 PMC9520817

[7] Shin H, Lee HS, Lee BC, Park G, Uranbileg K, Park Y, et al. The prevalence and clinical characteristics of borderline personality disorder in South Korea using National health insurance service customized database. Yonsei Med J. 2023;64(9):566–72. 37634633 10.3349/ymj.2023.0071 PMC10462810

[8] Paris J. Suicidality in borderline personality disorder. Med (Kaunas). 2019;55(6):223. 10.3390/medicina55060223 PMC6632023 31142033

[9] Meuldijk D, McCarthy A, Bourke ME, Grenyer BFS. The value of psychological treatment for borderline personality disorder: systematic review and cost offset analysis of economic evaluations. PLoS ONE. 2017;12(3):e0171592. 28249032 10.1371/journal.pone.0171592 PMC5332029

[10] Herpertz SC, Bertsch K, Jeung H. Neurobiology of criterion A: self and interpersonal personality functioning. Curr Opin Psychol. 2018;21:23–7. 28946053 10.1016/j.copsyc.2017.08.032

[11] van Zutphen L, Siep N, Jacob GA, Goebel R, Arntz A. Emotional sensitivity, emotion regulation and impulsivity in borderline personality disorder: a critical review of fMRI studies. Neurosci Biobehav Rev. 2015;51:64–76. 25616185 10.1016/j.neubiorev.2015.01.001

[12] Krause-Utz A, Winter D, Niedtfeld I, Schmahl C. The latest neuroimaging findings in borderline personality disorder. Curr Psychiatry Rep. 2014;16(3):438. 24492919 10.1007/s11920-014-0438-z

[13] Gunderson JG, Herpertz SC, Skodol AE, Torgersen S, Zanarini MC. Borderline personality disorder. Nat Rev Dis Primers. 2018;4:18029. 29795363 10.1038/nrdp.2018.29

[14] Soloff P, White R, Diwadkar VA. Impulsivity, aggression and brain structure in high and low lethality suicide attempters with borderline personality disorder. Psychiatry Res. 2014;222(3):131–9. 24656768 10.1016/j.pscychresns.2014.02.006 PMC4034388

[15] Holtmann J, Herbort MC, Wüstenberg T, Soch J, Richter S, Walter H, et al. Trait anxiety modulates fronto-limbic processing of emotional interference in borderline personality disorder. Front Hum Neurosci. 2013;7:54. 23459637 10.3389/fnhum.2013.00054 PMC3585713

[16] Sebastian A, Jung P, Krause-Utz A, Lieb K, Schmahl C, Tüscher O. Frontal dysfunctions of impulse control - a systematic review in borderline personality disorder and attention-deficit/hyperactivity disorder. Front Hum Neurosci. 2014;8:698. 25232313 10.3389/fnhum.2014.00698 PMC4153044

[17] Salehinejad MA, Ghanavati E, Rashid MHA, Nitsche MA. Hot and cold executive functions in the brain: A prefrontal-cingular network. Brain Neurosci Adv. 2021;5:23982128211007770. 10.1177/23982128211007769 PMC8076773 33997292

[18] Perez-Rodriguez MM, Bulbena-Cabré A, Bassir Nia A, Zipursky G, Goodman M, New AS. The neurobiology of borderline personality disorder. Psychiatr Clin North Am. 2018;41(4):633–50. 30447729 10.1016/j.psc.2018.07.012

[19] Schulze L, Schmahl C, Niedtfeld I. Neural correlates of disturbed emotion processing in borderline personality disorder: A multimodal Meta-Analysis. Biol Psychiatry. 2016;79(2):97–106. 25935068 10.1016/j.biopsych.2015.03.027

[20] Ruocco AC, Amirthavasagam S, Choi-Kain LW, McMain SF. Neural correlates of negative emotionality in borderline personality disorder: an activation-likelihood-estimation meta-analysis. Biol Psychiatry. 2013;73(2):153–60. 22906520 10.1016/j.biopsych.2012.07.014

[21] Donegan NH, Sanislow CA, Blumberg HP, Fulbright RK, Lacadie C, Skudlarski P, et al. Amygdala hyperreactivity in borderline personality disorder: implications for emotional dysregulation. Biol Psychiatry. 2003;54(11):1284–93. 14643096 10.1016/s0006-3223(03)00636-x

[22] Baczkowski BM, van Zutphen L, Siep N, Jacob GA, Domes G, Maier S, et al. Deficient amygdala-prefrontal intrinsic connectivity after effortful emotion regulation in borderline personality disorder. Eur Arch Psychiatry Clin Neurosci. 2017;267(6):551–65. 28039553 10.1007/s00406-016-0760-z PMC5561271

[23] Cristea IA, Gentili C, Cotet CD, Palomba D, Barbui C, Cuijpers P. Efficacy of psychotherapies for borderline personality disorder: A systematic review and Meta-analysis. JAMA Psychiatry. 2017;74(4):319–28. 28249086 10.1001/jamapsychiatry.2016.4287

[24] Zanarini MC, Frankenburg FR, Hennen J, Silk KR. Mental health service utilization by borderline personality disorder patients and axis II comparison subjects followed prospectively for 6 years. J Clin Psychiatry. 2004;65(1):28–36. 14744165 10.4088/jcp.v65n0105

[25] Bozzatello P, Rocca P, De Rosa ML, Bellino S. Current and emerging medications for borderline personality disorder: is pharmacotherapy alone enough? Expert Opin Pharmacother. 2020;21(1):47–61. 31693423 10.1080/14656566.2019.1686482

[26] Ripoll LH. Psychopharmacologic treatment of borderline personality disorder. Dialogues Clin Neurosci. 2013;15(2):213–24. 24174895 10.31887/DCNS.2013.15.2/lripoll PMC3811092

[27] Nitsche MA, Cohen LG, Wassermann EM, Priori A, Lang N, Antal A, et al. Transcranial direct current stimulation: state of the Art 2008. Brain Stimulat. 2008;1(3):206–23. 10.1016/j.brs.2008.06.004 20633386

[28] Nitsche MA, Fricke K, Henschke U, Schlitterlau A, Liebetanz D, Lang N, et al. Pharmacological modulation of cortical excitability shifts induced by transcranial direct current stimulation in humans. J Physiol (Lond). 2003;553(Pt 1):293–301. 12949224 10.1113/jphysiol.2003.049916 PMC2343495

[29] Nitsche MA, Doemkes S, Karaköse T, Antal A, Liebetanz D, Lang N, et al. Shaping the effects of transcranial direct current stimulation of the human motor cortex. J Neurophysiol. 2007;97(4):3109–17. 17251360 10.1152/jn.01312.2006

[30] Feeser M, Prehn K, Kazzer P, Mungee A, Bajbouj M. Transcranial direct current stimulation enhances cognitive control during emotion regulation. Brain Stimulat. 2014;7(1):105–12. 10.1016/j.brs.2013.08.006 24095257

[31] Konstantinou GN, Trevizol AP, Downar J, McMain SF, Vila-Rodriguez F, Daskalakis ZJ, et al. Repetitive transcranial magnetic stimulation in patients with borderline personality disorder: A systematic review. Psychiatry Res. 2021;304:114145. 34358761 10.1016/j.psychres.2021.114145

[32] Wang C, Zeng Q, Yuan Z, Wang W, Shen M. Effects of Low-Frequency (0.5 Hz) and High-Frequency (10 Hz) repetitive transcranial magnetic stimulation on neurological function, motor function, and excitability of cortex in ischemic stroke patients. Neurologist. 2023;28(1):11–8. 35452441 10.1097/NRL.0000000000000435 PMC9812416

[33] Reyes-López J, Ricardo-Garcell J, Armas-Castañeda G, García-Anaya M, Arango-De Montis I, González-Olvera JJ, et al. Clinical improvement in patients with borderline personality disorder after treatment with repetitive transcranial magnetic stimulation: preliminary results. Rev Bras Psiquiatr. 2018;40(1):97–104. 28614492 10.1590/1516-4446-2016-2112 PMC6899410

[34] Page M, McKenzie J, Bossuyt P, Boutron I, Hoffmann T, Mulrow CD, et al. The PRISMA 2020 statement: an updated guideline for reporting systematic reviews. BMJ. 2021;372:n71. 33782057 10.1136/bmj.n71 PMC8005924

[35] Cochrane. Handbook for Systematic Reviews of Interventions| Cochrane Training [Internet]. [cited 2024 Apr 10]. Available from: https://training.cochrane.org/handbook/current

[36] Rayyan [Internet]. [cited 2024 Aug 22]. Available from: https://rayyan.ai/.

[37] Higgins JPT, Altman DG, Gøtzsche PC, Jüni P, Moher D, Oxman AD, et al. The Cochrane collaboration’s tool for assessing risk of bias in randomised trials. BMJ. 2011;343:d5928. 22008217 10.1136/bmj.d5928 PMC3196245

[38] Reise SP, Moore TM, Sabb FW, Brown AK, London ED. The Barratt impulsiveness Scale-11: reassessment of its structure in a community sample. Psychol Assess. 2013;25(2):631–42. 23544402 10.1037/a0032161 PMC3805371

[39] Rodriguez-Seijas C, Thompson JS, Diehl JM, Zimmerman M. A comparison of the dimensionality of the Hamilton rating scale for anxiety and the DSM-5 Anxious-Distress specifier interview. Psychiatry Res. 2020;284:112788. 31978629 10.1016/j.psychres.2020.112788

[40] Jackson-Koku G. Beck depression inventory. Occup Med (Lond). 2016;66(2):174–5. 26892598 10.1093/occmed/kqv087

[41] Quilty LC, Robinson JJ, Rolland J-P, Fruyt FD, Rouillon F, Bagby RM. The structure of the Montgomery-Åsberg depression rating scale over the course of treatment for depression. Int J Methods Psychiatr Res. 2013;22(3):175–84. 24038301 10.1002/mpr.1388 PMC6878407

[42] Carrozzino D, Patierno C, Fava GA, Guidi J. The Hamilton rating scales for depression: A critical review of clinimetric properties of different versions. Psychother Psychosom. 2020;89(3):133–50. 32289809 10.1159/000506879

[43] Perez V, Barrachina J, Soler J, Pascual JC, Campins MJ, Puigdemont D, et al. The clinical global impression scale for borderline personality disorder patients (CGI-BPD): a scale sensible to detect changes. Actas Esp Psiquiatr. 2007;35(4):229–35. 17592784

[44] Zanarini MC, Vujanovic AA, Parachini EA, Boulanger JL, Frankenburg FR, Hennen J. Zanarini rating scale for borderline personality disorder (ZAN-BPD): a continuous measure of DSM-IV borderline psychopathology. J Pers Disord. 2003;17(3):233–42. 12839102 10.1521/pedi.17.3.233.22147

[45] de Wilde Brand O, Clarke S, Arntz A. The use of borderline personality disorder severity index-iv feedback in adjusting borderline personality disorder treatment: therapists and patients perspectives. BMC Psychiatry. 2022;22(1):469. 35836201 10.1186/s12888-022-04104-w PMC9284892

[46] Pfohl B, Blum N, St John D, McCormick B, Allen J, Black DW. Reliability and validity of the borderline evaluation of severity over time (BEST): a self-rated scale to measure severity and change in persons with borderline personality disorder. J Pers Disord. 2009;23(3):281–93. 19538082 10.1521/pedi.2009.23.3.281 PMC3608461

[47] GRADEpro [Internet]. [cited 2024 Apr 17]. Available from: https://www.gradepro.org/

[48] Calderón-Moctezuma AR, Reyes-López JV, Rodríguez-Valdés R, Barbosa-Luna M, Ricardo-Garcell J, Espino-Cortés M, et al. Improvement in borderline personality disorder symptomatology after repetitive transcranial magnetic stimulation of the dorsomedial prefrontal cortex: preliminary results. Rev Bras Psiquiatr. 2020;43(1):65–9. 32876128 10.1590/1516-4446-2019-0591 PMC7861182

[49] Feffer K, Lee HH, Wu W, Etkin A, Demchenko I, Cairo T, et al. Dorsomedial prefrontal rTMS for depression in borderline personality disorder: A pilot randomized crossover trial. J Affect Disord. 2022;301:273–80. 34942227 10.1016/j.jad.2021.12.038

[50] Cailhol L, Roussignol B, Klein R, Bousquet B, Simonetta-Moreau M, Schmitt L, et al. Borderline personality disorder and rTMS: a pilot trial. Psychiatry Res. 2014;216(1):155–7. 24503285 10.1016/j.psychres.2014.01.030

[51] Lisoni J, Miotto P, Barlati S, Calza S, Crescini A, Deste G, et al. Change in core symptoms of borderline personality disorder by tDCS: A pilot study. Psychiatry Res. 2020;291:113261. 32622171 10.1016/j.psychres.2020.113261

[52] Fu Y, Long Z, Luo Q, Xu Z, Xiang Y, Du W, et al. Functional and structural connectivity between the left dorsolateral prefrontal cortex and Insula could predict the antidepressant effects of repetitive transcranial magnetic stimulation. Front Neurosci. 2021;15:645936. 33841087 10.3389/fnins.2021.645936 PMC8032871

[53] Jin Y, Potkin SG, Kemp AS, Huerta ST, Alva G, Thai TM, et al. Therapeutic effects of individualized alpha frequency transcranial magnetic stimulation (alphaTMS) on the negative symptoms of schizophrenia. Schizophr Bull. 2006;32(3):556–61. 16254067 10.1093/schbul/sbj020 PMC2632240

[54] Benwell CSY, London RE, Tagliabue CF, Veniero D, Gross J, Keitel C, et al. Frequency and power of human alpha oscillations drift systematically with time-on-task. NeuroImage. 2019;192:101–14. 30844505 10.1016/j.neuroimage.2019.02.067 PMC6503153

[55] Wu M, Yu Y, Luo L, Wu Y, Gao J, Ye X, et al. Efficiency of repetitive transcranial direct current stimulation of the dorsolateral prefrontal cortex in disorders of consciousness: A randomized Sham-Controlled study. Neural Plast. 2019;2019:7089543. 31308848 10.1155/2019/7089543 PMC6594311

[56] De Vidovich GZ, Muffatti R, Monaco J, Caramia N, Broglia D, Caverzasi E, et al. Repetitive TMS on left cerebellum affects impulsivity in borderline personality disorder: A pilot study. Front Hum Neurosci. 2016;10:582. 27994543 10.3389/fnhum.2016.00582 PMC5136542

[57] Dębowska W, Więdłocha M, Dębowska M, Kownacka Z, Marcinowicz P, Szulc A. Transcranial magnetic stimulation and ketamine: implications for combined treatment in depression. Front Neurosci. 2023;17:1267647. 37954877 10.3389/fnins.2023.1267647 PMC10637948

[58] Eldaief MC, McMains S, Izquierdo-Garcia D, Daneshzand M, Nummenmaa A, Braga RM. Network-specific metabolic and haemodynamic effects elicited by non-invasive brain stimulation. Nat Ment Health. 2023;1(5):346–60. 37982031 10.1038/s44220-023-00046-8 PMC10655825

[59] Khan A, Wang X, Ti CHE, Tse C-Y, Tong K-Y. Anodal transcranial direct current stimulation of anterior cingulate cortex modulates subcortical brain regions resulting in cognitive enhancement. Front Hum Neurosci. 2020;14:584136. 33390917 10.3389/fnhum.2020.584136 PMC7772238

[60] Li Q, Fu Y, Liu C, Meng Z. Transcranial direct current stimulation of the dorsolateral prefrontal cortex for treatment of neuropsychiatric disorders. Front Behav Neurosci. 2022;16:893955. 35711693 10.3389/fnbeh.2022.893955 PMC9195619

[61] Hutton TM, Aaronson ST, Carpenter LL, Pages K, West WS, Kraemer C et al. The anxiolytic and antidepressant effects of transcranial magnetic stimulation in patients with anxious depression. J Clin Psychiatry. 2023;84(1). 10.4088/JCP.22m14571 36630648

