# Supplementary material for: Non-invasive brain stimulation for borderline personality disorder: a systematic review and network meta-analysis
Source: Ann Gen Psychiatry. 2025 Apr 16;24:24. doi: 10.1186/s12991-025-00561-1 (PMC12004652; doi:10.1186/s12991-025-00561-1)
Supplement: Supplementary file 1 — Supplementary Material 1 [file 12991_2025_561_MOESM1_ESM.docx]

**Online supplementary materials**

**Non-Invasive Brain Stimulation for Borderline Personality Disorder: A Systematic Review and Network Meta-Analysis**


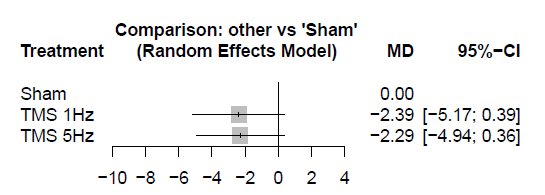


***Supplementary Figure S1.***  *Forest plot showing Abandonment which is a behavioral domain of CGI-BPD.*

*
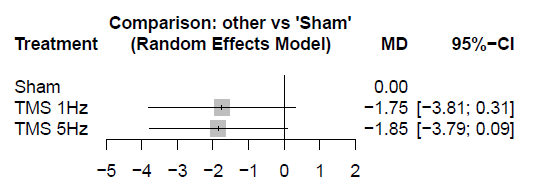
*

***Supplementary Figure S2.***  *Forest plot showing Affective instability which is a behavioral domain of CGI-BPD.*


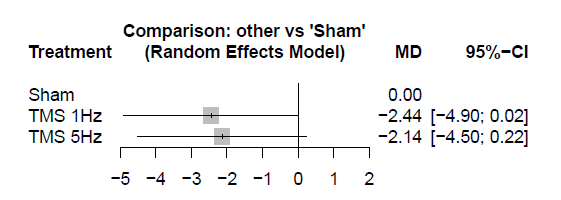


***Supplementary Figure S3.***  *Forest plot showing Anger which is a behavioral domain of CGI-BPD.*

*
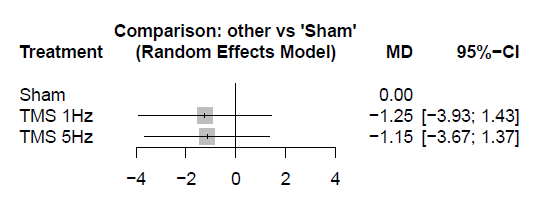
*

***Supplementary Figure S4.***  *Forest plot showing Emptiness which is a behavioral domain of CGI-BPD.*

*
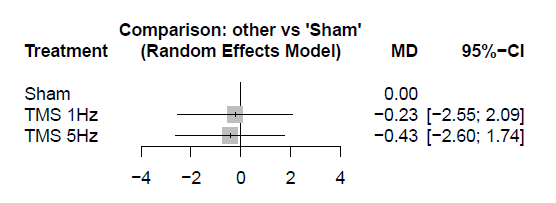
*

***Supplementary Figure S5.***  *Forest plot showing Identity which is a behavioral domain of CGI-BPD.*

*
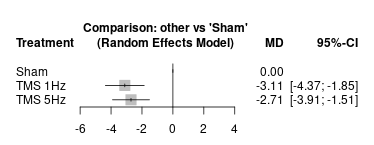
*

***Supplementary Figure S6.***  *Forest plot showing Impulsiveness which is a behavioral domain of CGI-BPD.*

*
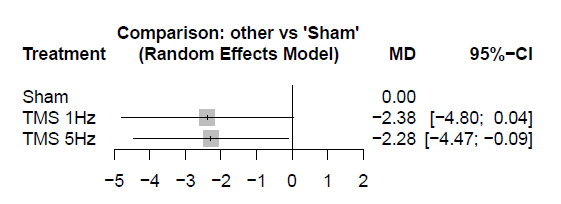
*

***Supplementary Figure S7.***  *Forest plot showing Paranoid ideation which is a behavioral domain of CGI-BPD.*

*
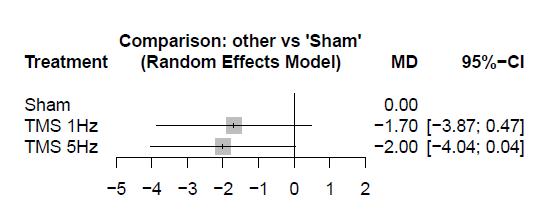
*

***Supplementary Figure S8.***  *Forest plot showing Unstable relationship which is a behavioral domain of CGI-BPD.*


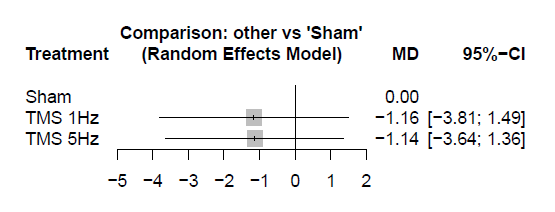


***Supplementary Figure S9.***  *Forest plot showing Suicidal ideation which is a behavioral domain of CGI-BPD.*


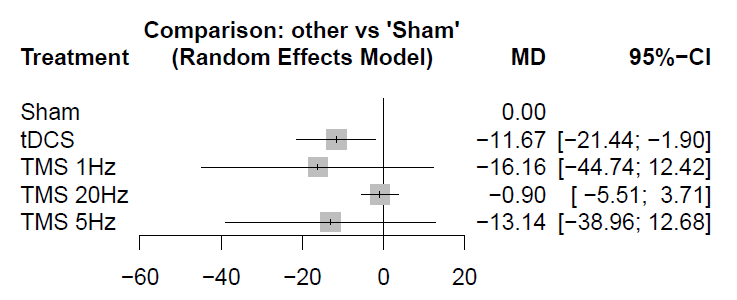


***Supplementary Figure S10.***  *Forest plot showing* ***BIS-11****.*


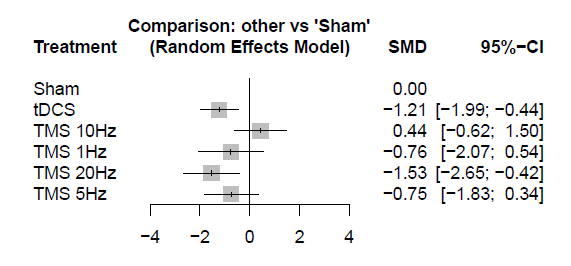


***Supplementary Figure S11.***  *Forest plot showing* ***depressive symptoms***

*
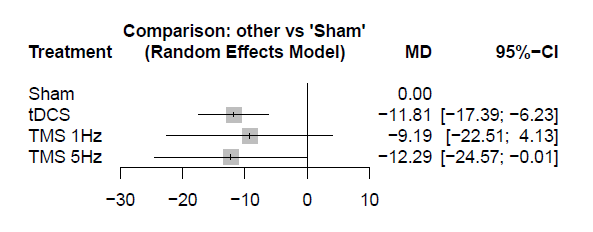
*

***Supplementary Figure S12.***  *Forest plot showing* ***HAM-A****.*

*
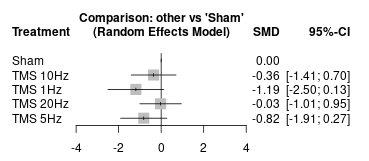
*

*
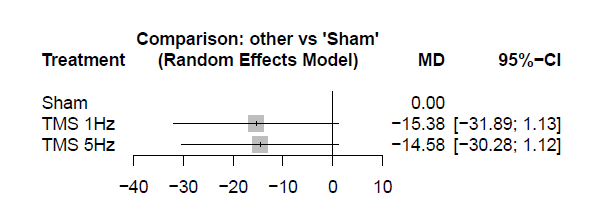
****Supplementary Figure S13.***  *Forest plot showing* ***BPD severity****.*

***Supplementary Figure S14.***  *Forest plot showing* ***CGI-BPD.***
